# A mechanical theory of competition between plant root growth and soil pressure reveals a potential mechanism of root penetration

**DOI:** 10.1038/s41598-023-34025-x

**Published:** 2023-05-09

**Authors:** Haruka Tomobe, Satoru Tsugawa, Yuki Yoshida, Tetsuya Arita, Allen Yi-Lun Tsai, Minoru Kubo, Taku Demura, Shinichiro Sawa

**Affiliations:** 1 Department of Civil and Environmental Engineering, Tokyo Institute of Technology, Kanagawa, 226-8502 Japan; 2 Faculty of Systems Science and Technology, Akita Prefectural University, Akita, 015-0055 Japan; 3 International Research Center for Agricultural and Environmental Biology (IRCAEB), Kumamoto University, Kumamoto, 860-8555 Japan; 4 Graduate School of Science and Technology, Kumamoto University, Kumamoto, 860-8555 Japan; 5 Center for Digital Green-innovation, Nara Institute of Science and Technology, Nara, 630-0192 Japan; 6 Division of Biological Science, Graduate School of Science and Technology, Nara Institute of Science and Technology, Nara, 630-0192 Japan

**Keywords:** Biophysics, Computational biophysics

## Abstract

Root penetration into the soil is essential for plants to access water and nutrients, as well as to mechanically support aboveground structures. This requires a combination of healthy plant growth, adequate soil mechanical properties, and compatible plant–soil interactions. Despite the current knowledge of the static rheology driving the interactions at the root–soil interface, few theoretical approaches have attempted to describe root penetration with dynamic rheology. In this work, we experimentally showed that radish roots in contact with soil of specific density during a specific growth stage fail to penetrate the soil. To explore the mechanism of root penetration into the soil, we constructed a theoretical model to explore the relevant conditions amenable to root entry into the soil. The theory indicates that dimensionless parameters such as root growth anisotropy, static root–soil competition, and dynamic root–soil competition are important for root penetration. The consequent theoretical expectations were supported by finite element analysis, and a potential mechanism of root penetration into the soil is discussed.

## Introduction

Plant roots push through the soil to access water and nutrients and provide mechanical support for aboveground organs of the plants. The conditions that regulate root penetration into the soil are thought to include the shape and growth of plant roots. For instance, the root cap plays an important role in penetration by secreting viscous mucilage and sloughing off its outer cells^1,2^, thus reducing the mechanical friction between the root tip and the soil. The shape of the root cap itself may also be a key factor, as the tip shape may be optimized by minimizing penetration stress while the root grows^3–6^. In addition to the primary root, the growth of lateral roots and root hairs may also play important roles during root penetration^7–9^. These results indicate that the morphology and dynamic growth of both primary roots and lateral structures (lateral roots and root hairs) are key factors in understanding how roots penetrate the soil.

On the other hand, the mechanical properties of the soil also affect to what extent roots can grow into the soil. One such factor is the void ratio of the soil, which correlates with the softness of the soil. Root penetration has been shown to be reduced in compacted soil with low void ratios^10–13^. Root penetration into the soil can be quantified through mechanical impedance, i.e., the penetration resistance the root encounters during growth^14–17^. Mechanical impedance can be measured as the force required to push a penetrometer probe through soil over the cross-sectional area of the penetrometer cone^14,15^. The mechanical impedance is thought to increase exponentially over time during root growth, as the void ratio decreases due to the soil being compacted by the growing root^18^. Recently, soil compactness was shown to affect root growth not only mechanically but also physiologically. Compact soil has been shown to restrict the diffusion of the gaseous plant hormone ethylene, which suppresses root elongation and promotes root thickening^19^. Moreover, the soil mechanical impedance can be affected by soil properties such as density and/or water content in the soil^20,21^. These lines of evidence indicate that both root growth and soil mechanical properties can be highly variable.

Despite the dynamic nature of soil and roots, the rheological mechanics of the plant–soil interface have yet to be described with mathematical models. An early attempt of this was made using continuum mechanics^22^, whereby the total penetration energy was estimated under the assumption that energy is conserved between the root and the soil. This revealed that the root–soil interface dictates the amount of energy required to break the surrounding soil. The mechanical impedance of the plant–soil interface has also been estimated by measuring the normal stress on the surface of a penetrometer cone, the cone semi-angle, and the coefficient of soil–object friction to approximate the behavior of a root^17,18^. Building on these pioneering studies, recent engineering approaches using the finite element method (FEM) have enabled the analyses of parameters dictating root penetration^23–25^. However, simple mathematical equations that reflect important root and soil parameters have not been investigated except in a recent work^26^.

Here, we examine the behaviors of radish roots at the very initial growth stage in soils with different mechanical properties, and determine the threshold of root penetration occurrence, which we call the root penetration criterion. This allowed us to identify conditions where roots fail to penetrate the soil. We then translated these conditions into equations describing how root penetration may respond to different soil types and then tested the resulting criterion with realistic plant–soil numerical simulations using FEM. Overall, we successfully summarized the plant–soil mechanical parameters into simple equations that will empower a systematic exploration of the root penetration criterion.

## Results

### Soil mechanical properties and root growth stages affect root penetration ability

**Figure 1:**
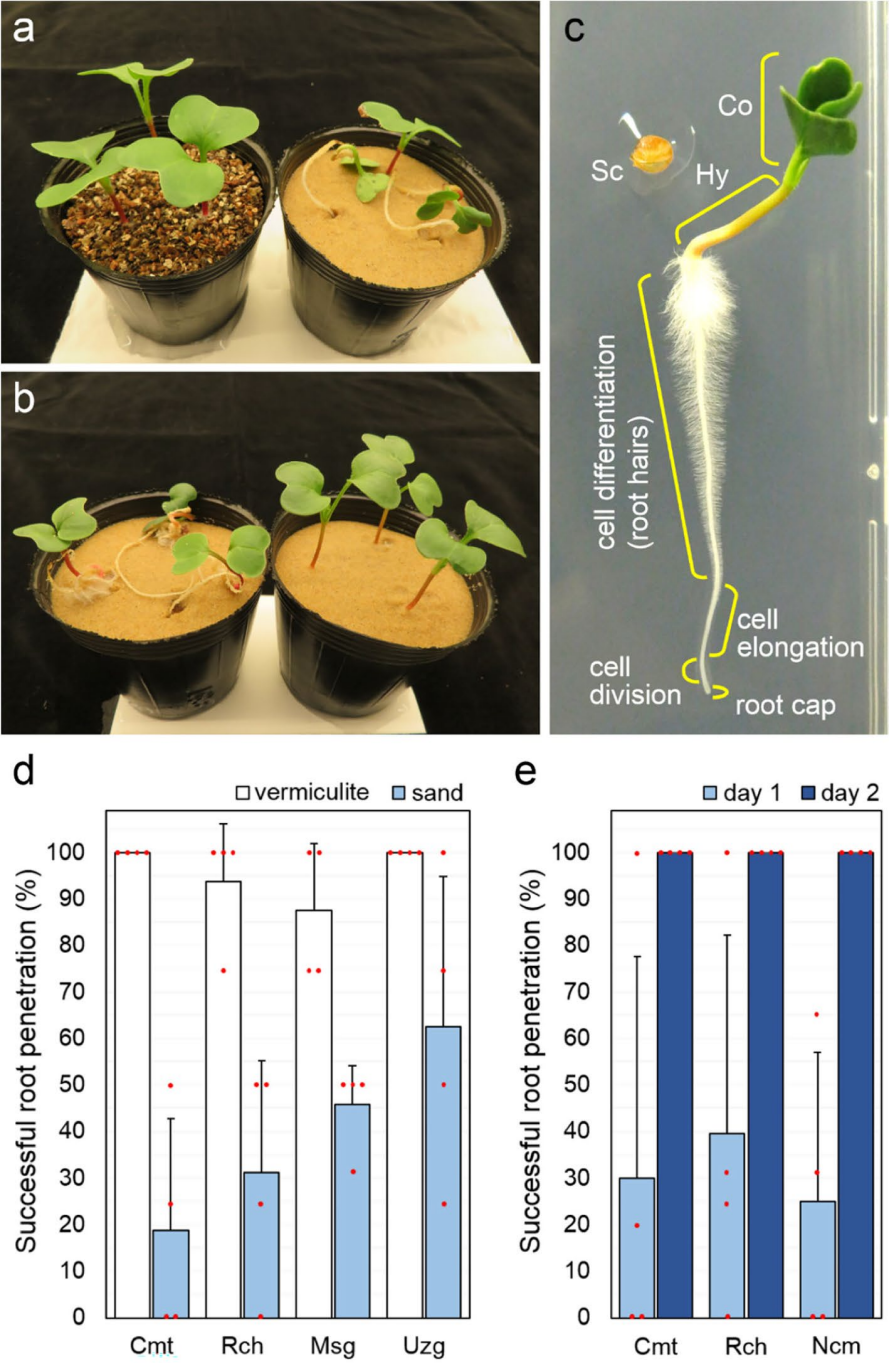
Radish seedling rooting behaviors are variable. (**a**–**c**) The penetration of radish roots into the soil is affected by the soil properties and seedling growth stage. (**a**) Representative images of 7-days after hydration (DAH) seedlings transferred to vermiculite (left) or silica sand (right) at 1-DAH. (**b**) Representative images of 7-DAH seedlings transferred to silica sand at 1-DAH (left) or 2-DAH (right). (**c**) A typical 4-DAH seedling grown on the surface of a vertically oriented agar plate. Co: cotyledons; Hy: hypocotyl; Sc: seed coat. Cell division is limited to the region near the root cap. (**d**) Rate of successful root penetration for two European and two Asian radish cultivars transferred to either vermiculite or silica sand at 1-DAH. Each data point is an average of four independent pots (n = 3–5 seedlings each). Error bars indicate standard deviation (SD). (**e**) Rate of successful root penetration in three European radish cultivars transferred to silica sand either at 1- or 2-DAH. Data show averages of four pots (n = 3–5 each) ± SD.

We observed that the roots of radish (*Raphanus sativus*) seedlings grown on silica sand^27^ frequently fail to penetrate the sand. Instead, they lifted the shoot part above the soil surface along with the upper region of the roots becoming exposed (Fig. 1a right). We confirmed that this root-uplifting phenomenon occurs in multiple radish cultivars (Comet, Red Chime, New Comet, Miyashige-Soubutori and Utsugi-Gensuke), as summarized in the supplementary material (Fig. 1d). Interestingly, these cultivars almost never exhibited root uplift when grown on coarse vermiculite (Fig. 1a left, 1d), suggesting that root penetration is dependent on soil mechanical properties. Silica sand and vermiculite have vastly different void ratios. Some soil samples with vermiculite have high void ratios of over 4.0^28^; in contrast, the silica sand used in this study has a void ratio of 0.95^29^. For seedlings with uplifted roots, we noticed sand grains could be found on the exposed root surface (Supplementary Fig. S2). Since root growth (cell division, elongation, and differentiation) is most pronounced near the root tip (Fig. 1c), the presence of sand grains suggests that these regions were once belowground and are being pushed up by cell elongation occurring at the root tip. We therefore hypothesized that this root-uplifting phenomenon may be caused by the failure of the root tip to penetrate the soil, possibly due to its low void ratio.

We consistently observed root uplift in radish seeds that had been hydrated on wet paper towels for 1 day before being transferred to sand (Supplementary Fig. S1a). At this stage, the seed coats have ruptured, while the radicles have initiated gravitropic bending but still lack root hairs (Supplementary Fig. S1b). In contrast, seeds that were allowed to germinate for 2 days showed longer (1–2 cm) primary roots with dense root hairs, as well as expanded green cotyledons (Supplementary Fig. S1c). Interestingly, these 2-day-old seedlings with longer roots did not exhibit root uplift when transferred to silica sand (Fig. 1b,e). The growth stage of the plant thus affects its ability to penetrate the soil, possibly due to the increased root–soil frictional forces exerted by the longer primary root with root hairs. These results suggested that seedlings with primary roots reaching a certain length with root hairs may provide better anchorage to promote soil penetration.

Clearly, root penetration potential is controlled by both the soil mechanical properties and the initial root length. Changes in these factors, such as fluctuating soil void ratios and root morphological changes during growth, all influence root penetration. This implies that competition likely occurs between these antagonizing forces at the root–soil interface.

### Theoretical formulation of the root penetration criterion in a linear regime

**Figure 2:**
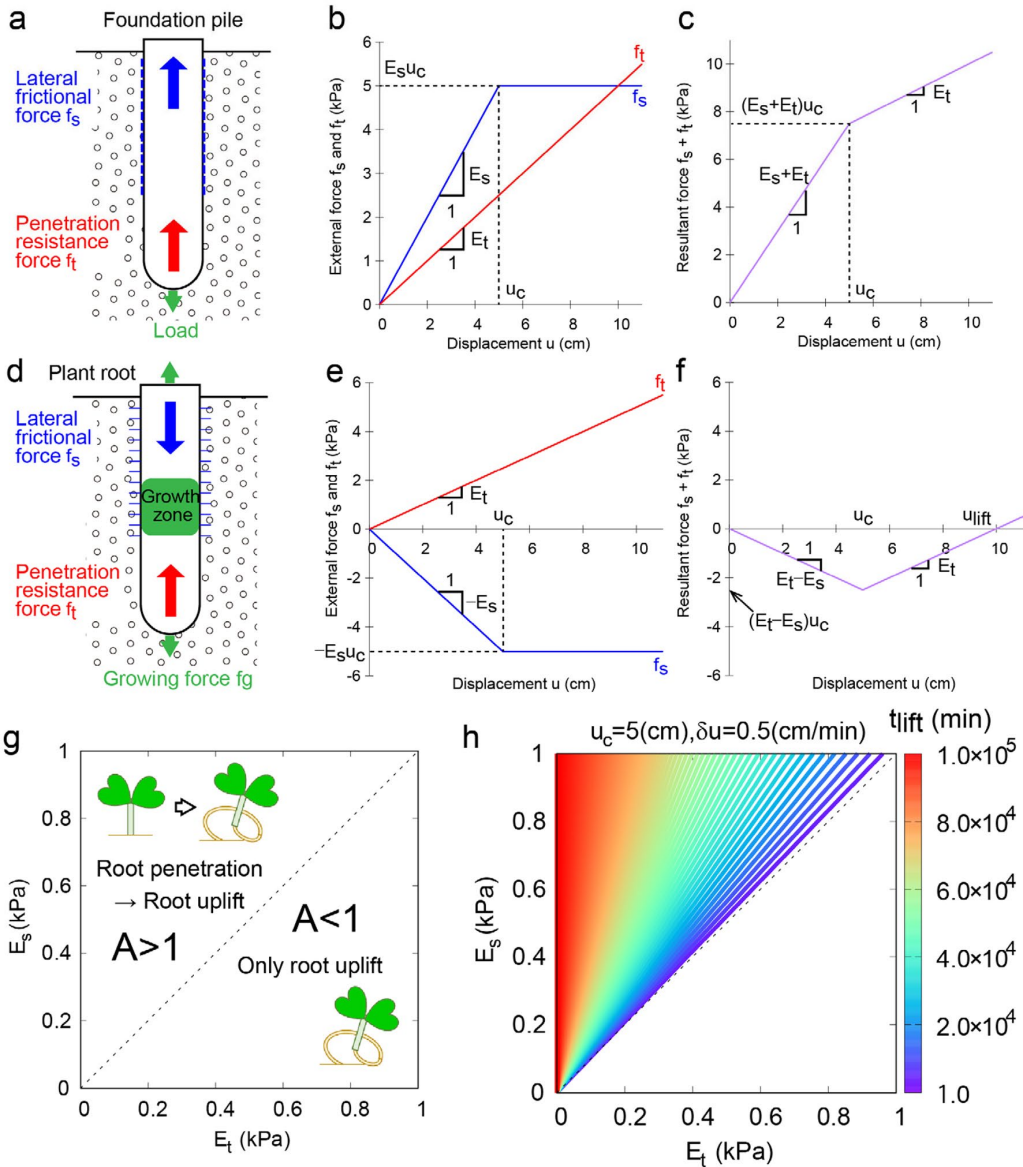
Theoretical evaluation of root penetration criterion in a linear regime. (**a**) Schematic diagram of the mechanical forces imposed on the foundation pile. (**b**) External force acting against the virtual displacement with lateral frictional force ${f}_{s}$ (blue) and penetration resistance force ${f}_{t}$ (red) as a function of displacement. (**c**) Resultant force acting against the virtual displacement for the foundation pile. (**d**) Schematic diagram of the mechanical forces imposed on the plant root. (**e**) External force acting against virtual displacement with lateral structures (lateral roots and root hairs) ${f}_{s}$ (blue) and penetration resistance force ${f}_{t}$ (red) as a function of displacement. (**f**) Resultant force acting against virtual displacement for the plant root. (**g**) Root penetration criterion in a linear regime. (h) Color plot of the time interval before root uplift ${t}_{lift}$.

The root-uplifting phenomenon prompted us to develop a theoretical framework to better understand the factors shaping root penetration. To understand the phenomenon of root uplift, we focused especially on the very early stage of plant roots in the transplanting situation hereafter. To develop a general description for plant–soil mechanics, we considered the well-studied mechanical forces acting on foundation piles (Fig. 2a)^30,31^ as an example to understand plant–soil interactions. We note the definitions of the following variables and parameters are listed in Table 1. The forces acting on foundation piles consist of three components: lateral frictional force ${f}_{s}$ derived from the object–soil interface; penetration resistance force ${f}_{t}$, derived from the reaction force at the tip of the object; and downward load. For simplicity, we assumed that ${f}_{s}$ is proportional to the upward virtual displacement (infinitesimal change) of the root base with coefficient ${E}_{s}$, and ${f}_{t}$ is proportional to the downward virtual displacement of the root tip with coefficient ${E}_{t}$. The vertical axis was set to be positive in an upward direction. In the case of pile penetration, only the downward virtual displacement at the tip of the pile was expected, inducing ${f}_{s}$ and ${f}_{t}$ in an upward direction as illustrated in Fig. 2b. It should be noted here that this example of lateral friction has the slip threshold displacement ${u}_{c}$, beyond which the friction is restricted to a constant due to object slippage. With this setup, the resultant force is described as the sum of ${f}_{s} + {f}_{t}$, where both forces act as the bearing capacity or the penetration resistance to the load downward (Fig. 2c).

**Table 1:** List of definitions of all the parameters.

Variable/parameter	Definition
${f}_{s}$	Lateral frictional force
${f}_{t}$	Penetration resistance force
${f}_{s}+{f}_{t}$	Resultant force of penetration and friction
${f}_{g}$	Growing force of the root
${E}_{s}$	Elastic coefficient associated with ${f}_{s}$
${E}_{t}$	Elastic coefficient associated with ${f}_{t}$
$\varphi$	Wall extensibility
${u}_{c}$	Threshold of displacement for root to slip
${u}_{lift}$	Threshold of displacement for root to lift up
$\delta u$	Strain associated with the unit time scale
${t}_{lift}$	Time for root to lift up
$u(+)$	Virtual displacement of the root base
$u(-)$	Virtual displacement of the root tip
${G}_{p}$	Primary growth rate of the root
${G}_{s}$	Secondary growth rate of the root
$L(t)$	Length of the zone presenting root hairs
${L}_{0}$	Initial length of the zone presenting root hairs
$t$	Time variable
$u(\pm )$	Virtual displacement $\mathrm{u}(+)$ or $\mathrm{u}(-)$
$R(t)$	Radius of the root
${R}_{0}$	Initial radius of the root
${u}_{s}$	Effective displacement of the frictional domain
${P}_{0}$	Initial earth pressure
${P}_{p}$	Preconsolidation stress
$P^{\prime}(={P}_{p}-{P}_{0})$	Relative soil pressure
${P}_{t}$	Current soil pressure
${e}_{0}$	Initial void ratio
${e}_{t}$	Current void ratio
$\uplambda$	Normal consolidation coefficient
${V}_{0}$	Initial void volume
${V}_{t}$	Current void volume
${V}_{s}$	Soil volume
${D}_{v}$	Distance from the depth at which the soil does not move when the root grows
$S$	Soil mechanical parameter
$\alpha$	Non-dimensional parameter representing growth anisotropy of the root
$\beta$	Non-dimensional parameter representing ratio between ${P}_{p}$ and ${E}_{s}$
$\gamma$	Non-dimensional parameter representing ratio between $S$ and ${G}_{s}$
$E$	Young’s modulus of the soil

In contrast to foundation piles, the forces acting on a plant root are of a different scenario. As the growing force within the root ${f}_{g}$ are exerted both upward and downward, frictional forces are induced by the lateral roots and root hairs near the ground in a downward direction (Fig. 2d). Under these circumstances, the negative ${f}_{s}$ competes with the positive ${f}_{t}$ (Fig. 2e). The virtual displacement can be interpreted as ${f}_{g}/\varphi$ in both upward and downward directions, where the index $\varphi$ refers to the wall extensibility, which represents the growth capacity as the cell wall is loosened under turgor pressure. Therefore, the direction of the actual displacement can be upward when the resultant force is positive, or downward when the force is negative. We illustrate the typical root behavior in Fig. 2f, where the resultant force becomes negative at the beginning of growth, before becoming positive after it reaches the threshold (defined as ${u}_{lift}$) when the condition ${f}_{s} + {f}_{t}=0$ is met. We define the degree of competition between the root and the soil by the index $A={E}_{s}/{E}_{t}$. As such, the root penetrates the soil only when $A>1$ (Fig. 2g). The time interval before the root uplift is dictated by ${t}_{lift}={Au}_{c}/\delta u$, where $\delta u$ (cm/min) is the strain associated with the unit time scale. As shown in Fig. 2h, ${t}_{lift}$ increased gradually under the assumption ${u}_{c}=5$ cm and $\delta u=0.5$ cm/min, indicating that, in the reddish region, only root penetration is observed over a typical experimental time scale (~ minutes or ~ hours) less than the order of ${10}^{4}$ min.

Under a linear regime, the root penetration criterion therefore simply reflects the degree of competition between the root and the soil. Root uplift will take place only when the force exerted by the soil outcompetes the force exerted by the root.

### Theoretical formulation of the root penetration criterion in a nonlinear regime

Next, we sought to more accurately describe the mechanical terms of ${f}_{s}$ and ${f}_{t}$. According to the above discussion, the plant undergoes these two antagonizing forces; therefore, we first define each effect as the positive virtual displacement of the root base $u(+)$ and the negative virtual displacement of the root tip $u(-)$ along the vertical axis. Briefly, we extended the model in terms of the growth anisotropy of the plant root and the competition between lateral friction and soil compression stress outlined below.

**Figure 3:**
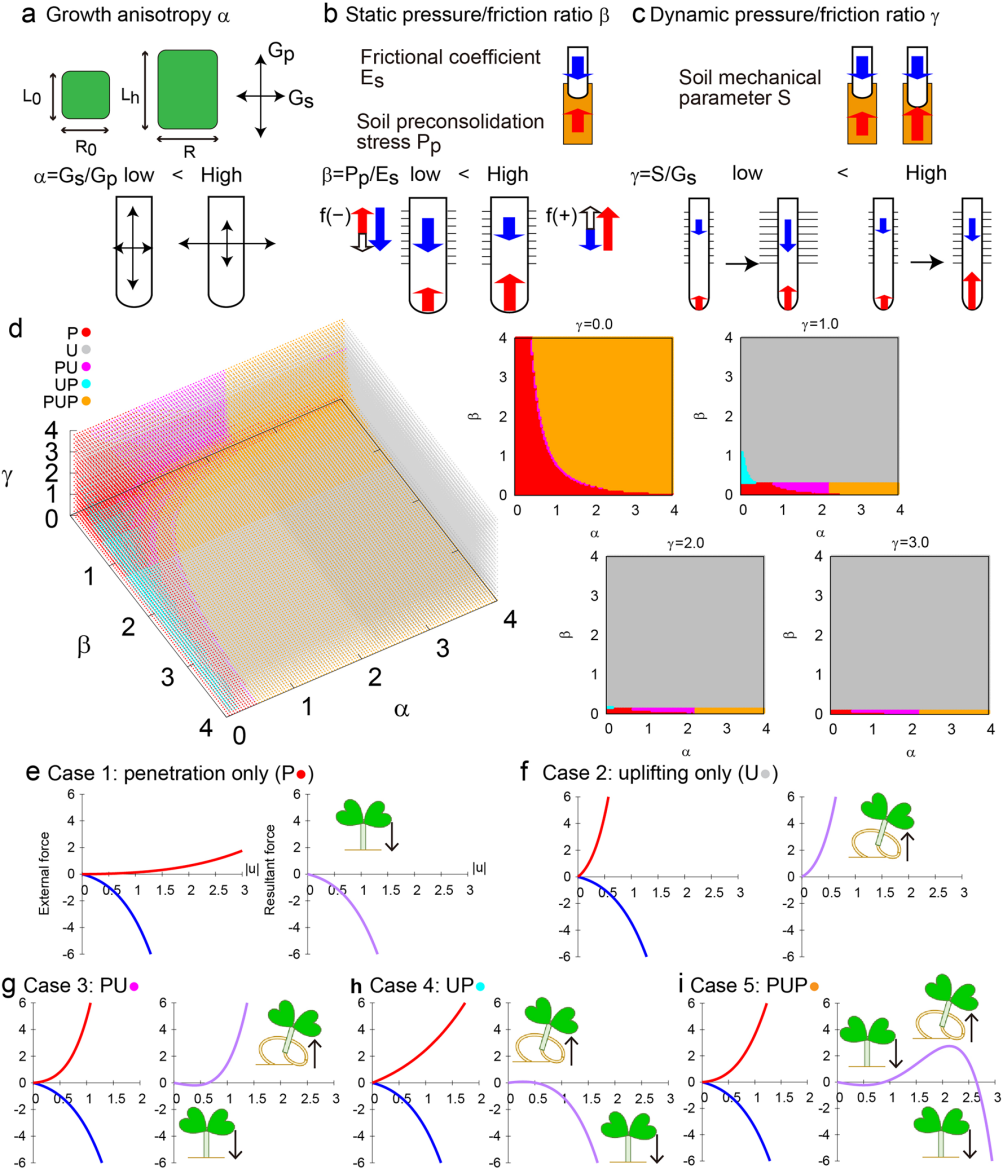
Theoretical evaluation of root penetration criterion in a nonlinear regime. (**a-c**) Schematic diagram of the growth anisotropy (**a**), static pressure and friction ratio (**b**) and dynamic pressure and friction ratio (**c**). (**d**) Color plot of the diffrent cases depending on the indices of α, β, and γ. (**e**–**i**) External force and resultant force as a function of the absolute value of the virtual displacement. Case 1: penetration only (P) (**e**). Case 2: uplifting only (U) (**f**). Case 3: P, then, U (**g**). Case 4: U, then P (**h**). Case 5: P, then, U, then P (**i**).

Given the plant growing force = ${f}_{g}/\varphi$, let us consider the growth anisotropy of the plant root (Fig. 3a). For the following discussion, we assume that plant primary growth is linear with a growth rate ${G}_{p}$ and that secondary growth is also linear with a growth rate ${G}_{s}$ such that the length of the zone presenting root hairs $L\left(t\right)$ is defined as:

1 $$L\left( t \right) = L_{0} + G_{p}t,$$

where $t=u(\pm )/\delta u$, and the radius of the root is defined as

2 $$R\left(t\right)={R}_{0} + {G}_{s}t.$$

Since the lateral frictional force ${f}_{s}$ should depend on the primary growth $L\left(t\right)$ due to the enlargement of the frictional area, and on the coefficient ${E}_{s}$ and the effective displacement of the frictional domain ${u}_{s}$, ${f}_{s}$ can be written as

3 $${f}_{s}= -L\left(t\right){E}_{s}{u}_{s}.$$

The force ${f}_{s}$ may not have to be proportional to the virtual displacement $u(+)$ because the frictional force becomes strong when the lateral structure increases. Therefore, we assumed the effective displacement is a nonlinear function, i.e., ${u}_{s}=\mathrm{exp}\left(\frac{{G}_{s}u\left(+\right)}{\delta u}\right)-1$ using the secondary growth rate ${G}_{s}$. Thus the force ${f}_{s}$ can be rewritten as,

4 $${f}_{s}= -\left({L}_{0}+\frac{{G}_{p}u\left(+\right)}{\delta u}\right){E}_{s}\left(\mathrm{exp}\left(\frac{{G}_{s}u\left(+\right)}{\delta u}\right)-1\right),(u(+)\le {u}_{c})$$

5 $${f}_{s}= -\left({L}_{0}+\frac{{G}_{p}u\left(+\right)}{\delta u}\right){E}_{s}\left(\mathrm{exp}\left(\frac{{G}_{s}{u}_{c}}{\delta u}\right)-1\right),(u(+)>{u}_{c}).$$

Due to the presence of both the intrinsic frictional force and the root growing force, ${f}_{s}$ becomes a nonlinear function of $u(+)$. We note that the assumption of linear growth with an exponential function can be modified to the other growth trend^32^.

For ${f}_{t}$, we considered the contribution of the root cap cross-section area to the compression of the soil cylinder below the root (Fig. 3b). As the soil at the early stage of penetration is assumed to be of normal density, the initial earth pressure ${P}_{0}$ with the relative soil pressure $P^{\prime}(={P}_{p}-{P}_{0})$ with the preconsolidation stress ${P}_{p}$ (soil weight × depth), and the current soil pressure ${P}_{t}$ are defined with the initial void ratio ${e}_{0}$ and the current void ratio ${e}_{t}$ as:

6 $$\frac{{e}_{t}-{e}_{0}}{\mathrm{ln} {P}_{t}- \mathrm{ln} ({P}^{^{\prime}}+{P}_{0})}= - \lambda ,$$

where $\lambda$ is the normal consolidation coefficient^18^. This relationship indicates how the temporal change in soil pressure between ${P}_{0}$ and ${P}_{t}$ affects the relative change in the soil void ratio ${e}_{0}$ and ${e}_{t}$. To relate this to the displacement $\mathrm{u}(-)$, we need to know the relationship between the void ratio and the void volume, as the void ratio is defined as ${e}_{t}={V}_{t}/{V}_{s}$, ${e}_{0}={V}_{0}/{V}_{s}$ with the soil volume ${V}_{s}$, the initial void volume ${V}_{0}$ and the current void volume ${V}_{t}$. Using the virtual displacement $u\left(-\right)$, we formulated the difference between ${V}_{0}$ and ${V}_{t}$ as follows:

7 $${V}_{0}-{V}_{t} = u\left(-\right)\pi R{\left(t\right)}^{2}.$$

We assume the following two physical constraints: (a) the equilibrium between the root volume expansion and the soil volume reduction associated with root growth, and (b) the equilibrium between the root growing force and the soil compaction force. Using the parameter ${D}_{v}$ as the depth at which the soil does not move when the root grows, we got the following relationship.

8 $${D}_{v}\pi R{\left(t\right)}^{2}={V}_{s}+{V}_{0}.$$

Using Eqs. (7) and (8), the displacement $u(-)$ can be rewritten as

9 $$u\left(-\right)={D}_{v}\frac{{e}_{0}-{e}_{t}}{{e}_{0}+1}.$$

Thus, the soil pressure at time t leads to

10 $${P}_{t}={P}_{p}\mathrm{ exp }\left(\frac{u\left(-\right)\left({e}_{0}+1\right)}{\lambda {D}_{v}}\right).$$

Therefore, the penetration resistance force can be rewritten as

11 $${f}_{t}= \pi {\left({R}_{0} +\frac{{G}_{s}u\left(-\right)}{\delta u}\right)}^{2}{P}_{p}\left(\mathrm{exp} \left(Su\left(-\right)\right)-1\right),$$

where the soil mechanical parameter $S$ is defined as $S=\frac{{e}_{0}+1}{\lambda {D}_{v}}.$ Due to the intrinsic penetration resistance force and the root growing force, ${f}_{t}$ is also a nonlinear function of $u(-)$.

With these formulations, the mechanics of root penetration are thus evaluated as the competition between ${f}_{s}$ and ${f}_{t}$ in a nonlinear manner. If the root penetrates the soil ($|{f}_{t}|>|{f}_{s}|$), then a positive shear force $f(+)={f}_{t}$ is applied. Conversely, if the root fails to penetrate the soil ($|{f}_{s}|>|{f}_{t}|$), then a negative shear force $f(-)={f}_{s}$ is applied (Fig. 3b). The governing equation is written as follows (using $\mathrm{\delta u}=1$ for simplicity):

$$F\left({L}_{0},{R}_{0},{G}_{p},{G}_{s},{E}_{s},{P}_{p},S\right)={f}_{s}+{f}_{t}$$

12 $$F\left({L}_{0},{R}_{0},{G}_{p},{G}_{s},{E}_{s},{P}_{p},S\right)=-\left({L}_{0}+{G}_{p}u\right){E}_{s}\left(\mathrm{exp}\left({G}_{s}u\right)-1\right)+\pi {\left({R}_{0}+{G}_{s}u\right)}^{2} {P}_{p} \left(\mathrm{exp} \left(Su\right)-1\right),\left(u\le {u}_{c}\right)$$

13 $$F\left({L}_{0},{R}_{0},{G}_{p},{G}_{s},{E}_{s},{P}_{p},S\right)= -\left({L}_{0}+{G}_{p}u\right){E}_{s}(\mathrm{exp}({G}_{s}{u}_{c})-1)+\pi {\left({R}_{0}+{G}_{s}u\right)}^{2}{P}_{p} (\mathrm{exp}(Su)-1). (u>{u}_{c}).$$

Contrary to the linear regime, the time duration of force application is important for the nonlinear regime, and there are a few more nondimensional parameters related to the time duration. We introduce the nondimensional growth anisotropy parameter $\alpha$ with the relationship ${G}_{s}=\alpha {G}_{p}$. We introduce the nondimensional parameter $\beta$, which describes the relative contribution of the static soil parameter ${P}_{p}$ to the frictional coefficient ${E}_{s}$ $\left(\beta ={P}_{p}/{E}_{s}\right)$ and the other non-dimensional parameter $\gamma$, which describes the relative contribution of the dynamic soil mechanical parameter to the secondary growth $(\gamma =S/{G}_{s})$. As the parameter $S$ indicates the soil mechanical property after time interval t, the nondimensional parameter $\gamma$ means the competition between dynamic soil change and root growth in radius (Fig. 3c). To explore the typical behaviors of $\alpha$, $\beta$, and $\gamma$, we made the following substitutions: ${E}_{s}=1, {G}_{p}=1,{G}_{s}=1,{L}_{0}=1, \; \mathrm{and} \; {R}_{0}=1.$

14 $$F\left(\alpha ,\beta ,\gamma ,u\right)=-\left(1+u\right)\left(\mathrm{exp}\left(u\right)-1\right)+\pi \beta {\left(1+\alpha u\right)}^{2}\left(\mathrm{exp}\left(\gamma u\right)-1\right),(u\le {u}_{c})$$

15 $$F\left(\alpha ,\beta ,\gamma ,u\right)=-\left(1+u\right)\left(\mathrm{exp}\left(u\right)-1\right)+\pi \beta {\left(1+\alpha u\right)}^{2}\left(\mathrm{exp}\left(\gamma u\right)-1\right),\left(u>{u}_{c}\right).$$

We named this formula the root penetration criterion. The root is predicted to penetrate the soil if $F\left(\alpha , \beta , \gamma , u\right)>0$, while the root is predicted to fail soil penetration and lift up the seedling if $F\left(\alpha , \beta , \gamma , u\right)<0$. To investigate whether the root successfully penetrates the soil, we employed the Newton–Raphson method to numerically detect solutions for the equation $F(\alpha , \beta , \gamma , u)=0$ (Fig. 3d). With the three dimensionless parameters growth anisotropy $\alpha$, soil-pressure/friction relativity $\beta$, and soil/lateral-root relativity $\gamma$ (Fig. 3c), we constructed a simplified diagram to illustrate how these parameters change in the different cases below. We defined five cases describing the root dynamics over a given time scale ($t<{u}_{c}/\delta u$) for the penetration state (P-state) and the uplift state (U-state) as a function of the parameters $\alpha$, $\upbeta$, and $\upgamma$.

- Case 1: P only: $F(\alpha ,\beta ,\gamma ,0)>0$ without solutions for $u>0$ (Fig. 3e)
- Case 2: U only: $F(\alpha ,\beta ,\gamma ,0)<0$ without solutions for $u>0$ (Fig. 3f)
- Case 3: P, then U: $F(\alpha ,\beta ,\gamma ,0)>0$ with one solution for $u>0$ (Fig. 3g)
- Case 4: U, then P: $F(\alpha ,\beta ,\gamma ,0)<0$ with one solution for $u>0$ (Fig. 3h)
- Case 5: P, then U, then P: $F(\alpha ,\beta ,\gamma ,0)>0$ with two solutions for $u>0$ (Fig. 3i)

Note that the UPU state was not numerically detected due to the quadratic form in front of the exponential term in ${f}_{t}$.

The root penetration criterion in the nonlinear regime is therefore described by the resultant force $F(\alpha ,\beta ,\gamma ,0)$, and the competition between soil and root depends on the mechanical forces ${f}_{s}$ and ${f}_{t}$, the time duration of the applied forces, and the intrinsic plant growth.

### Validation of the effects of ${\varvec{\beta}}$ by the finite element method

**Figure 4:**
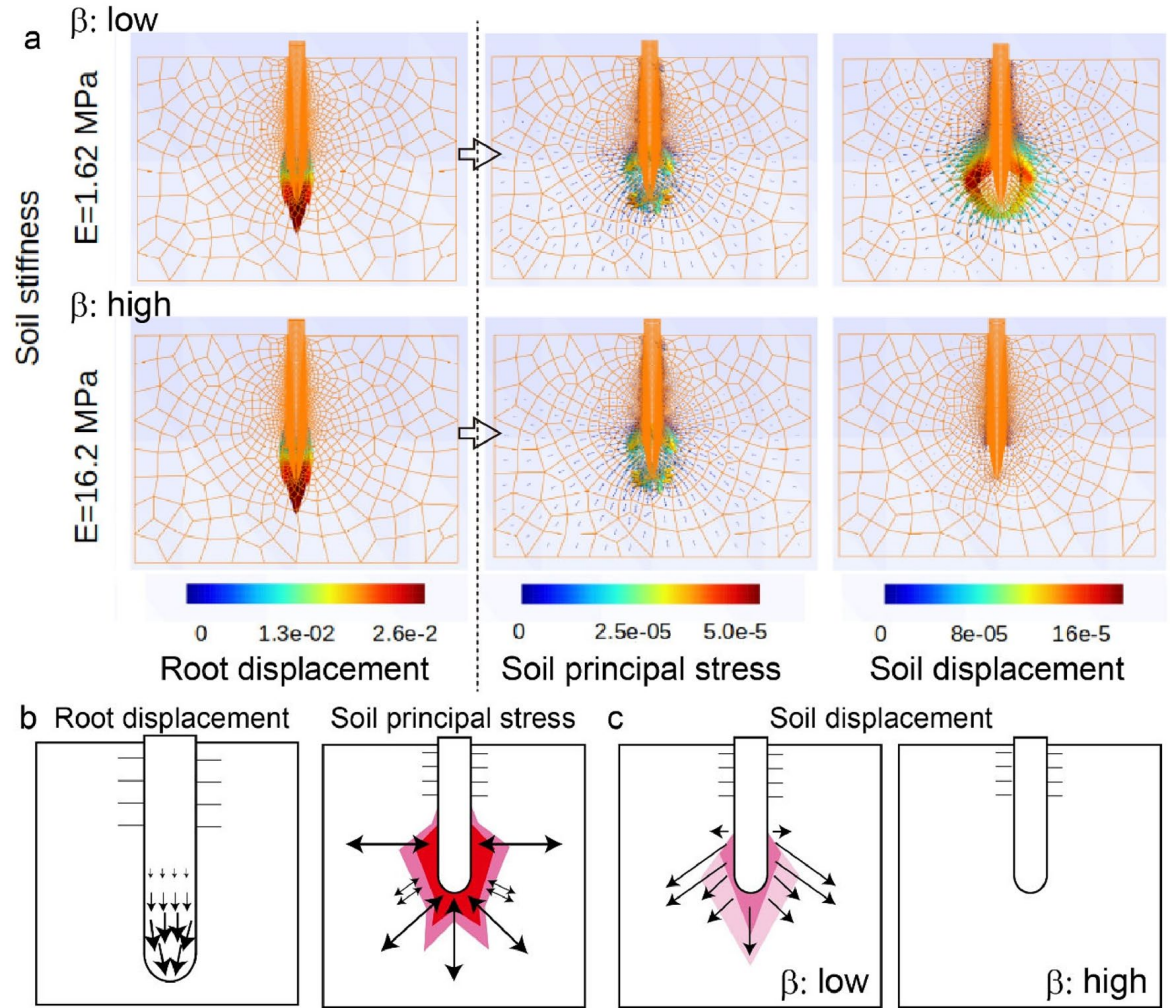
Results of root penetration numerical analysis using the contact finite element method. (**a**) Root displacement, soil principal stress and soil displacement of the simulations for two β-values by changing soil strength E (kPa). The units of color codes are cm (left), MPa (middle), and cm (right). From the initial displacement (left), the resulting stress and displacement after deformation were calculated (right). (**b**) Schematic illustrations of root displacement and soil principal stress. (**c**) Schematic illustrations of soil displacement for low β and for high β.

As the frictional force ${f}_{s}$ can be modulated by simulations using the finite element method, we tested the effects of varying $\beta$ values. We note that we focused only on the validation of the parameter $\beta$ because of computationally challenging bottlenecks to introduce an appropriate boundary condition with soil under anisotropic growth for $\mathrm{\alpha }$ and additional dynamic cohesion between root and soil for $\gamma$. To establish the forces acting on the root and soil, we set the root in a fixed position and observed small displacements and changes in mechanical stresses in soil of different frictional coefficients. Specifically, Young’s modulus of the soil changed to ${E = 1.62\,(\text{MPa})}$ for lower $\upbeta$ and to ${E = 1.62\,(\text{MPa})}$ for higher $\beta$. According to the nonlinear equations above, the soil displacement should decrease with greater $\beta$. With the same initial root displacement (left panels in Fig. 4a), we validated that the principal stress of the soil is almost the same regardless of $\beta$ values, while soil displacement correlates with $\beta$ (Fig. 4b,c). If the same root displacement is assigned at the tip (Fig. 4b left), the obtained soil principal stress would then be concentrated in two regions around the growth area near the root tip for both $\beta$ values (Fig. 4b right). We also noticed that the mechanical stresses near the root growth region are higher in the lateral direction, due to drastic friction increases in the lateral region. Soil displacement negatively correlates with $\beta$ values (soil stiffness) (Fig. 4c). In fact, soil displacements for high $\beta$ values were reduced to almost zero, indicating that the soil does not move in response to root displacement.

We conclude that root penetration becomes more likely with decreasing $\beta$ values, which is consistent with our theoretical estimation.

## Discussion

Here, we constructed a unifying formula describing the root penetration criterion, especially for the very early stage of plant roots in the transplanting situation, with three dimensionless parameters: root growth anisotropy α, static root–soil competition $\beta$, and dynamic root–soil competition $\gamma$. Our findings indicate that the root has two intrinsically antagonistic responses during soil penetration: growth of the primary root ($\mathrm{\alpha }$) and growth of the lateral roots and root hairs ($\upbeta$ and $\upgamma$).

This observation raises the question of whether the growth of each root type affects the other. For example, it is possible that both primary and secondary growth of roots are functions of soil mechanical properties, implying that the parameter $\mathrm{\alpha }$ might be a function of $\upbeta$ and $\upgamma$. Conversely, the parameters $\upbeta$ and $\upgamma$ can be affected by the plant growth anisotropy $\mathrm{\alpha }$. This may be addressed by testing whether primary root growth inhibits lateral structure growth, and vice versa. The relationship between primary and lateral roots may be related to the correlation between the mechanical stresses on the two root types. Importantly, our unifying theory may allow a more systematic exploration of the allometric relationship between root width and length^33^ and predicting the longest mechanically sustainable root as well as the highest height of trees, as in ref^34^. These parameters are essential for the quantitative characterization of root–soil mechanics.

The root uplifting phenomenon observed in this study indicates that growth conditions can influence root development; however, the biological relevance of root-lifting behavior remains unclear. This question may be related to the diversity of root penetration ability within and across plant species. The abilities of plant roots to penetrate soil likely vary between species; for example, sorghum has been shown to successfully penetrate silica sand where radish cannot^27^. Within one species, we observed variations in soil penetrating activities among the radish cultivars tested, implying that root growth mechanics are divergent even within a single species (Fig. 1d). These results may point to a genetic regulation of root growth mechanics, in addition to the abovementioned dimensionless parameters.

Moreover, we obtained two important results from our simulations using the finite element method. First, the principal stress of the soil indicated that the pressure exerted by the lateral organs in the root elongation zone pushes the soil aside to facilitate root elongation (Fig. 4b). We applied growth stress up to 1.0 MPa in the present simulation to remain consistent with previous studies^24,35^. According to Coulomb's law of friction^36^, the highest possible estimated friction is approximately 0.6 MPa. Such large friction becomes comparable to the mechanical impedance experienced at the root tip, which prevents any movement of the root tip with high $\beta$. Second, the displacement of the soil near the root tip exhibits a bifurcated pattern on either side of the root (Fig. 4c). Since soil is resilient against compressive stress but fragile against tensile stress, this bifurcation may more efficiently separate the soil to allow the root to elongate.

For future work in experiments, observation of the soil mechanical properties associated with root uplift will be required. Figure S4a shows the dry density and the moisture content of the soil utilized in the experiment, respectively. Consequently, the void ratio were estimated as 1.03 and 12.2 for the sand and for the vermiculite, respectively. Figure S4b shows that the soil environment is kept constant during the experiment, and that no drastic change in the soil structure is emerged. Considering the results in Fig. 1d, this indicates that the lower the void ratio is, the higher the pot penetration success rate is, which is consistent with the theoretical evaluation based on our formulation (Fig. 3).

For future work in theory, the validity of the $\mathrm{\alpha }$- and $\gamma$-indices will be evaluated separately through improved FEM simulations with the growth of lateral roots. For instance, it has been reported that hormones such as ethylene affect root girth^19,37,38^, which is directly associated with the dimensionless parameter α. It may be interesting to couple the present model with chemical analysis with ethylene to determine the relationship between the mechanics and the physiology of root growth. More specifically, the range of $\mathrm{\alpha }$ between 0.0 and 2.0 will be important to validate this model since the range contains three different penetration modes. Furthermore, it is known that soil density and water potential influence soil mechanical impedance^20,21^. Hence, it will also be interesting to investigate the effects of these factors against dimensionless parameters in our model.

Finally, our research framework may be applicable not only to plant biology but also to different fields of research such as plant phenotyping and biomimetics. Although our finding of the root uplift associated with the plant growth state and soil mechanical properties might be phenomenologically trivial, sequential experiments and theoretical formulation of the root uplift with validation by a mechanical model should be a powerful tool to find the mechanical characteristics of plant roots. For example, our approach to evaluate the parameter set (${\upalpha }$, $\upbeta$, $\upgamma$) for different types of plants may become a new approach to regulate the plant with a mechanical perspective, in a similar way to a genetic regulation of root hairs^39^. As demonstrated in our mechanical simulation, the primary root pushes the soil aside in a bifurcated manner as it grows downward, thereby adjusting the void ratio and the soil strength to favor root elongation. This hidden mechanical knowledge may be a key concept to construct root-inspired foundation piles in the field of biomimetics^40,41^.

## Methods

### Plant materials and experimental conditions

The radish (*Raphanus sativus*) cultivars used in this study are listed in Supplementary Table S1. All cultivars are of neither wild origin nor rare preserved genetic resources. The seeds are mass-produced by major commercial seed producers, and can be purchased from local markets in Japan. All plant experiments were carried out in accordance with relevant guidelines. For soil culture, radish seeds were hydrated on wet paper towels at 25 °C for 1 or 2 days. Germinated seedlings of comparable sizes were transferred to plastic pots (width 6 cm, height 5.5 cm, volume 130 mL) filled with either vermiculite (Ohishi Bussan Co. Ltd., Japan) or silica sand (Toyoura standard sand, Toyoura Keiseki Kogyo K.K., Japan)^42,43^. Details are shown in Supplementary Fig. S1. The soil water retention curves of these materials are shown in Alowaisy’s experiments^44^. Three to five seedlings were grown per pot. The rates of root uplift (defined as seedlings with at least 1 cm of the main root exposed to the air) were calculated for each pot, and then the averages for four pot cultivars were calculated. For growth on agar plates shown in Fig. 1c, seeds were surface-sterilized with diluted sodium hypochlorite solution and sown onto plates containing 1 × Murashige and Skoog (MS) medium with 0.75% (w/v) agar and 1.5% (w/v) sucrose, with the pH adjusted to pH 5.8. All pots and plates were cultivated in growth chambers at 22 °C under continuous white LED illumination.

### Finite element analysis

The numerical analysis scheme is essentially based on ref 25. The growth stress model was obtained by adding a new momentum term to account for the growth stress according to the momentum conservation law. The constitutive model for soil and plants was constructed based on the Neo-Hookean model. Young's modulus and Poisson's ratio are the same as for the roots and soil in ref 25. Here, the Young's modulus of the root was set to 35.0 MPa, and Poisson's ratio for both the root and the soil were set to 0.30. In addition, the Young's modulus of the soil was set to 1.62 MPa for soft soil, and 16.2 MPa for hard soil. The friction coefficient was set to 0.60 according to ref 21, and the root–soil contact surface was given a cohesion coefficient of 28.0 kPa to represent the cohesion caused by root hairs.

The finite element mesh of the soil and the roots is shown in Supplementary Fig. S3a. The mesh was automatically generated in a 2D space using the modified Delauney triangular division implemented by the Gmsh software^45^ to create a finer mesh structure around the roots, where large deformations were expected. Here, the root and soil meshes were defined separately, and the contact elements were automatically generated at the root–soil interface during the analysis. As shown in Supplementary Fig. S3, the roots and the soil were defined separately, with the growth area defined at the tip of the roots. Growth stress was generated in this growth region responsible for root elongation. To guarantee a viable solution for the governing equation, it was necessary to fix the upper part of the root as described in the main text. In contrast, the root cap was only in contact with the soil and was not fixed; thus, it was free to move in response to the forces between the root and the soil. If the root and soil moved downward at the same time, penetration was considered to have occurred. If the soil did not move, penetration was considered to have failed. It should be noted that it is possible to quantitatively compare the dimensionless parameters measured in the FE analysis with the root penetration criterion in the Eqs. (14) and (15), which is difficult in many experiments.

## Supplementary Information

Supplementary Information.

## Data Availability

The datasets used and/or analysed during the current study are available from the corresponding author on reasonable request.
