# Supplementary material for: A mechanical theory of competition between plant root growth and soil pressure reveals a potential mechanism of root penetration
Source: Sci Rep. 2023 May 9;13:7473. doi: 10.1038/s41598-023-34025-x (PMC10170176; doi:10.1038/s41598-023-34025-x)
Supplement: Supplementary file 1 — Supplementary Information. [file 41598_2023_34025_MOESM1_ESM.docx]

**
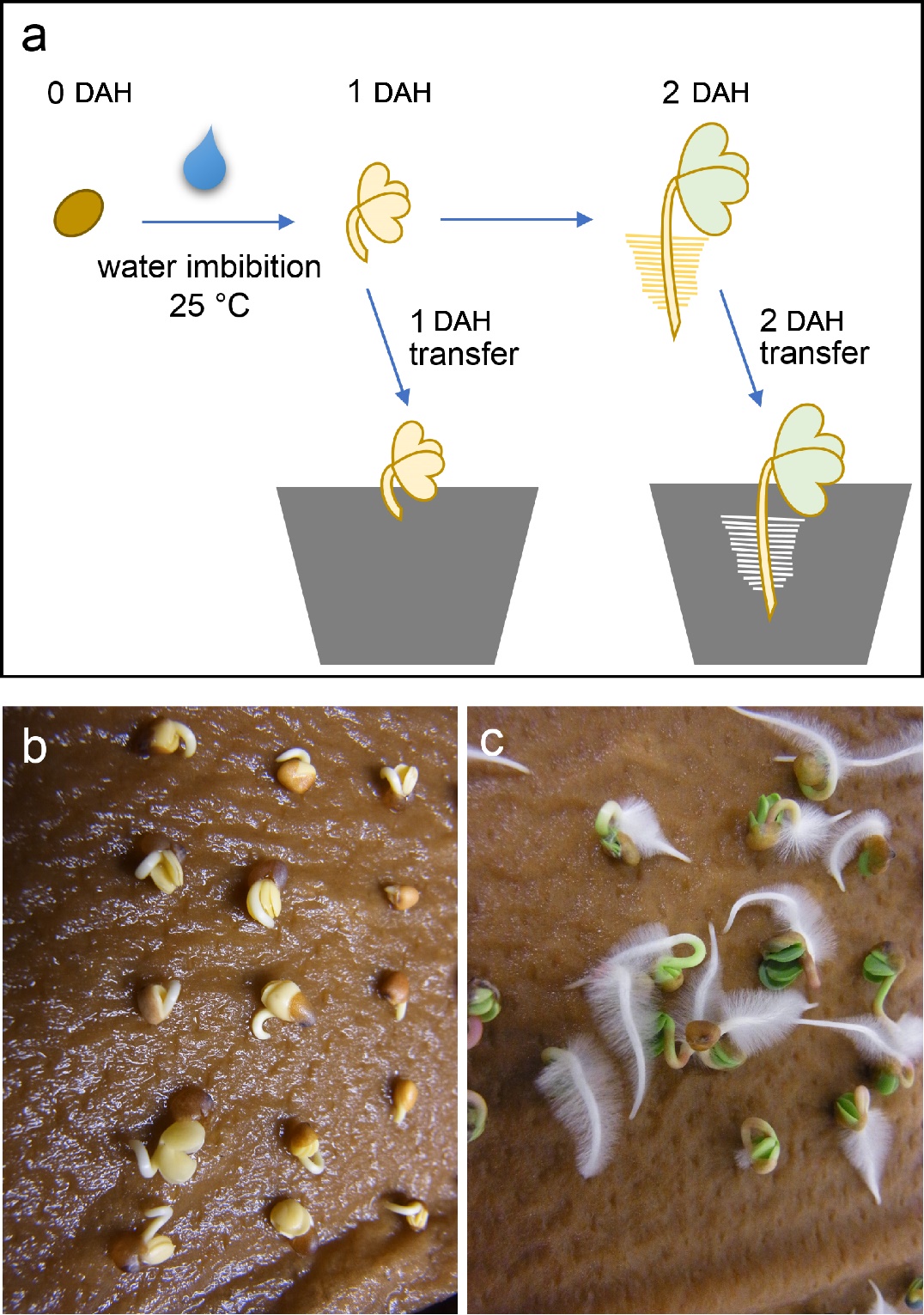
 Supplementary Figure S1.** Principle of the radish root penetration assay. (a) Schematic diagram of the radish root penetration assay. DAH, Days after hydration.

(b, c) Representative 1-DAH (b) and 2-DAH (c) seedlings germinated on a wet paper towel and transferred to pots at the indicated time points. Seedlings of comparable sizes are selected and transferred to soil or sand, as illustrated in (a).


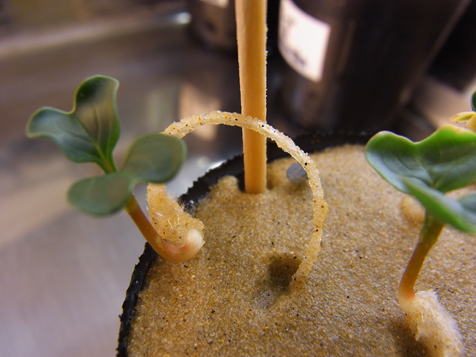


**Supplementary Figure S2.** Close-up of a radish seedling with exposed root that failed to penetrate silica sand. Note the root surface is covered with sand grains.

**
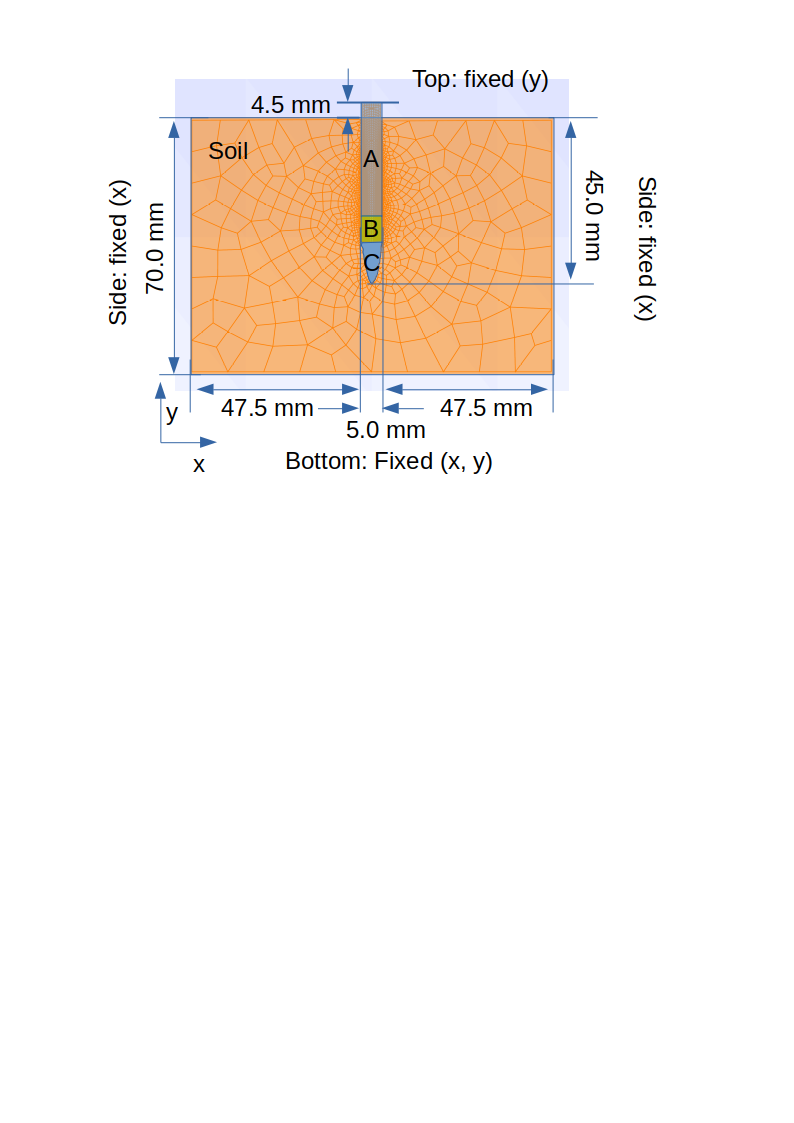
**

**Supplementary Figure S3.** Mesh and boundary conditions for finite element method (FEM) analysis. Note that the top of the root domain is fixed and growth stress is present in B.


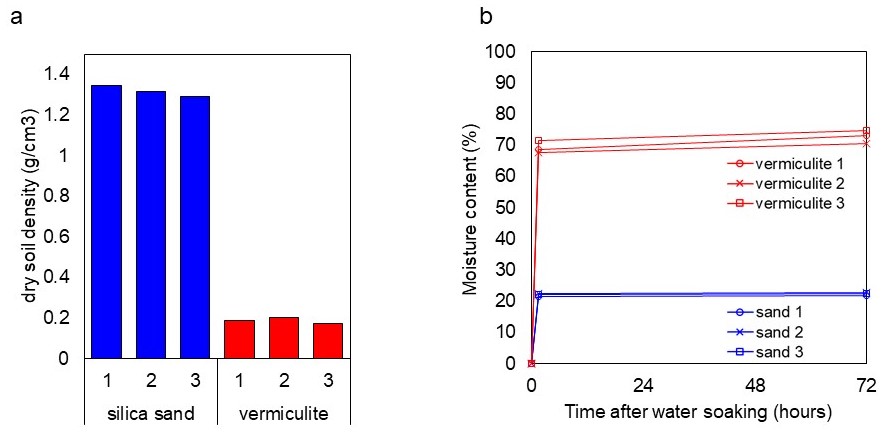


**Supplementary Figure S4.** (a)Dry soil density for silica sand and for vermiculite. (b) Moisture content for silica sand and for vermiculite as a function of time after water soaking (hours).

Supplementary Table S1. Radish (*Raphanus sativus*) cultivars used in this study

| Full Name | Code | Species | Variety | Source |
| --- | --- | --- | --- | --- |
| Comet | Cmt | *Raphanus sativus* | Red globe radish (European) | Daiso Industries, Hiroshima, Japan |
| Red Chime | Rch | *Raphanus sativus* | Red globe radish (European) | Sakata Seed, Yokohama, Japan |
| New Comet | Ncm | *Raphanus sativus* | Red globe radish (European) | Takii Seed, Kyoto, Japan |
| Miyashige-Soubutori | Msg | *Raphanus sativus* | Daikon (Asian) | Daiso Industries, Hiroshima, Japan |
| Utsugi-Gensuke | Uzg | *Raphanus sativus* | Daikon (Asian) | Daiso Industries, Hiroshima, Japan |

Supplementary Table S2. Material parameters used in this study and references

| Material | Parameter name | Value | Unit | Reference |
| --- | --- | --- | --- | --- |
| Soil | Young’s modulus | *1.62* | MPa | Tomobe 2019 |
|  | Poisson’s ratio | *0.3* |  | Tomobe 2019 |
| Root | Young’s modulus | *35.0* | MPa | Bizet 2016 |
|  | Poisson’s ratio | 0.3 |  | Tomobe 2019 |
|  | Pressure | 1.0 | MPa | Clark 1996; Dupuy 2018 |
| Interface | Friction coefficient | 0.6 |  | Tomobe 2019 |
|  | Cohesion | *28.0* | kPa | Tomobe 2021 |
